# Effects of Ivabradine on Residual Myocardial Ischemia after PCI Evaluated by Stress Echocardiography

**DOI:** 10.1155/2019/9185876

**Published:** 2019-04-01

**Authors:** Simone Calcagno, Fabio Infusino, Olga Dettori, Temistocle Taccheri, Pasqualina Bruno, Viviana Maestrini, Gennaro Sardella, Massimo Mancone, Francesco Fedele

**Affiliations:** Cardiovascular, Respiratory, Geriatric, Anesthesiologic and Nephrologic Sciences Department, Umberto I Hospital, Sapienza University of Rome, Rome, Italy

## Abstract

**Background:**

Residual angina after PCI is a frequently occurring disease. Ivabradine improves symptoms but its role in patients without left ventricular systolic dysfunction is still unclear. The aim was to quantify the effects of ivabradine in terms of MVO_2_ indicators and diastolic function.

**Methods:**

Twenty-eight consecutive patients with residual angina after PCI were randomized to ivabradine 5 mg twice/day (IG) or standard therapy (CG). All patients performed a stress echocardiography at the enrollment and after 30 days. MVO_2_ was estimated from double product (DP) and triple product (TP) integrating DP with ejection time (ET). Diastolic function was evaluated determining E and A waves, E′ measurements, and E/E′ ratio both at rest and at the peak of exercise.

**Results:**

The exercise time was longer in IG 9′49″ ± 48″ vs 8′09″ ± 59″ in CG (*p*=0.0001), reaching a greater workload (IG 139.3 ± 13.4 vs CG 118.7 ± 19.6 Watts; *p*=0.003). MVO_2_ expressed with DP and TP was significantly higher in IG (DP: IG 24194 ± 2697 vs CG 20358 ± 4671.8, *p*=0.01; TP: IG 17239 ± 4710 vs CG 12206 ± 4413, *p*=0.007). At peak exercise, the ET was diminished in IG than CG. The analysis of diastolic function after the exercise revealed an increase of E and A waves, without difference in the E/A ratio. The E′ wave was higher in IG than CG, and in the same group, the differences between baseline and peak exercise were greater (∆E′3.14 ± 0.7 vs 2.4 ± 1.13, *p*=0.047). The E/E′ ratio was reduced in patients treated with ivabradine (IG 10.2 ± 2.0 vs CG 7.9 ± 1.6, *p*=0.002).

**Conclusions:**

Ivabradine seems to produce a significant improvement of ischemic threshold, chronotropic reserve, and diastolic function.

## 1. Introduction

Stable angina is a syndrome characterized by a transitory condition of acute myocardial ischemic attacks caused by an imbalance between myocardial perfusion and metabolic demand [1]. The antianginal drugs and the percutaneous coronary revascularization are essential to treat the persistent symptoms. However, in some cases, when small vessels or side branches are significantly involved with hard or impossible reperfusion, drug therapy is the only strategy to prevent the worsening of ischemic heart disease and to reduce the symptoms. Heart rate is the major determinant of cardiac output and myocardial oxygen consumption; therefore, the reduction of the heart rate in patients with stable angina can be considered a goal of the therapy [2]. Ivabradine is an antianginal agent that specifically inhibits the pacemaker (If) current, resulting in selective HR reduction with no negative effects on blood pressure and inotropism [3]. In patients with chronic heart failure, ivabradine improves clinical outcomes if used with beta blockers under well-documented efficacy and safety [4].

In previous studies, ivabradine reduced anginal symptomatology but has not clearly demonstrated improvements of the outcomes in patients with chronic ischemic disease without left ventricular systolic dysfunction [5–7]. More recently, in CONTROL-2 Study, in patients with stable angina, the combination of the two therapies ivabradine and *β*-blockers together demonstrated good tolerability, safety, and also pronounced clinical improvements, compared to *β*-blockers up-titration [8]. Moreover, an interesting SHIFT substudy showed the reversed LV remodeling with marked reductions of LV volumes and significant improvements of LV ejection fraction (LVEF) in patients treated with ivabradine [9].

In this context, in order to define the role of ivabradine in patients with residual angina after PCI, we aimed to quantify the clinical benefits of adding ivabradine to standard anti-ischemic therapy, in symptomatic patients for residual myocardial ischemia after PCI, using more precise indicators of oxygen consumption.

Furthermore, we studied the effects of ivabradine on diastolic function (both at rest and after exercise) and on the ventricular remodeling.

## 2. Methods

We investigated the efficacy of treatments with ivabradine in addition to full anti-ischemic therapy (as per guidelines) compared with the latter alone in patients with signs or symptoms of residual angina underwent percutaneous coronary intervention plus stent implantation. The main purpose was to evaluate if addition of ivabradine to standard therapy might increase the threshold for angina improving the stress tolerance and exercise duration in terms of double product (DP) and triple product (TP), respectively, calculated as a product between HR and systolic blood pressure and the product between DP and ejection time. In our opinion these, indirectly, reflect the true myocardial oxygen consumption (MVO_2_) and the improvement of mechanical load that the ventricle can withstand at different levels of exercise. Triple product is closely related to the tension-time index, a measure of ventricular work and oxygen demand that is found by multiplying the average pressure in the ventricle during the period in which it ejects blood by the time it takes to do this [10].

The second objective was to evaluate changes at rest and after the stress test of diastolic function and ventricular remodeling, using echocardiographic parameters.

### 2.1. Study Population

In this randomized, prospective, single-center study, all the patients selected were with chronic coronary artery disease undergoing percutaneous coronary intervention (PCI) plus stent implantation, residual angina, and on treatment with full anti-ischemic therapy with and without ivabradine. In all patients, revascularization was complete as far as possible, all coronary lesions resulting in a stenosis greater than 50% in vessels of a caliber greater than two millimeters were treated. The inclusion criteria were coronary artery disease with chronic stable angina for more than three months (Canadian Cardiovascular Society–CCS-class I–III); percutaneous revascularization with stent implantation at least one; signs/symptoms of residual ischemia; sinus rhythm; HR ≥ 70 bpm at rest; ability to perform an echocardiogram stress test with the tilting bicycle stress test (BST); good acoustic window; and age ≥ 18 years. The main exclusion criteria were drugs intolerance or hypersensitivity, EF ≤ 40% with NYHA class III to IV; CCS IV; atrial fibrillation or flutter; presence of a pacemaker or implantable defibrillator; II or III degree AV block; HR ≤ 70 bpm at rest or sick sinus syndrome; any condition that could interfere with the ability to exercise stress test such as Wolff–Parkinson–White syndrome, left bundle branch block, left ventricular hypertrophy; rate-corrected QT interval (QTc) greater than 500 ms or the use of drugs that prolong the QTc interval; symptomatic hypotension or uncontrolled hypertension (systolic blood pressure at rest ≥ 180 mmHg or diastolic blood pressure ≥ 100 mmHg); severe liver disease and severe renal impairment (creatinine clearance ≤ 30 ml/min); electrolyte disorders; and uncontrolled thyroid disease and pregnancy. All patients signed informed consent prior to randomization. The study was designed according to the principles outlined in the Declaration of Helsinki and was approved by the Ethics Committee and Institutional Review.

### 2.2. Study Design and Treatment

All patients with CCS I–III angina were considered, and only patients who met the inclusion criteria, after signing the informed consent form, were included in our study. The treatment was assigned on the basis of a 1 : 1 ratio to receive ivabradine 5 mg twice daily (ivabradine group, IG) or standard therapy according to the guidelines (control group, CG). Both therapeutic strategies were titrated to the maximum tolerated dose, in particular *β*-blockers. All patients were submitted to clinical evaluation and exercise stress echocardiography at enrollment time (T1) and after 30 days of therapy (T2). During the period between exercise stress tests, clinical evaluation with ivabradine therapy up-titration was performed after 15 days.

Patient evaluation included physical examination, HR measurement by 12-lead electrocardiograms (ECG), two-dimensional, Doppler and Tissue Doppler echocardiography, using Philips iE33 system (Andover, Massachusetts, USA) and a supine bicycle ergometer stress test. During the test, operators recorded echocardiograms before and during the exercise. The parameters measured were left ventricular (LV) 2D diameters, systolic function parameters, ejection time, LV end-systolic and end-diastolic volumes in relation to body surface area (end-diastolic and end-systolic indices (EDI and ESI)), and LV ejection fraction (LVEF) assessed according to Simpson's method (as suggested by the American Society of Echocardiography and European Association of Echocardiography) [11].

#### 2.2.1. Bicycle Stress Test

Exercise test was performed using a semirecumbent and tilting bicycle ergometer (X-SCRIBE EKG Analysis, Mortara Instruments; Ergometrics 800s, Ergoline, West-Germany) with an initial workload set at 25 Watt and increments of 25 Watt/2 min. HR and rhythm is continuously recorded using a 12-lead electrocardiogram; blood pressure was measured at baseline, at peak exercise, and during the last minute of each stage including recovery.

The rate of exercise, which was measured in metabolic equivalents (1 metabolic equivalent = 3.6 ml/kg/min), and the duration of exercise were assessed as well.

Chronotropic reserve was estimated using the following formula: 100 × (peak HR − resting HR)/(220 − age) − resting HR.

Stress test was interrupted if the patient developed chest pain, ST segment elevation >0.1 mV at 80 ms from the J point, or a significant adverse event (significant ventricular arrhythmia, limiting breathlessness, dizziness, muscular exhaustion, chest pain, arterial pressure drop ≥ 10 mmHg with symptoms, or severe systemic hypertension).

#### 2.2.2. Echocardiography

Images were acquired in standard views and displayed side by side in a quad-screen format. All images were digitally recorded in continuous-loop format. Total work at the ischemic threshold and peak exercise was calculated. Double product (DP) was calculated during the last stage of exercise performed by multiplying maximum systolic BP by maximum HR; triple product (TP) was obtained integrating DP with ejection time (ET) measured with mitral annular PW-TDI (BP × SBP × ET).

In addition, diastolic function was evaluated by PW Doppler E and A waves, TDI-derived E′ measurements, and E/E′ ratio. Mitral annular E′ velocity was estimated as the average between lateral and septal velocity.

Drugs with possible interactions with ivabradine such as nondihydropyridine calcium-channel blockers, class I antiarrhythmics, and strong inhibitors of cytochrome P450 3A4 were not allowed, whereas short-acting nitrates were allowed up to 3 hours before exercise or after exercise if needed.

At the end of the tests, double and triple products and diastolic function evaluation results were collected by a blind operator.

All parameters recorded and calculated in off-line analysis were included in our register.

### 2.3. Statistical Analysis

The sample size was not calculated because it was a pilot study. Continuous variables were presented as mean ± standard deviation (SD) and categorical variables as percentages. Categorical variables were compared among groups using the chi-square test or Fisher's exact test when appropriate, whereas continuous variables were compared with Student's *t*-test. All tests were two-sided, and a *p* value less than 0.05 was considered statistically significant. All analyses were performed using SPSS software version 20.0 for Windows.

## 3. Results

**Figure 1:**
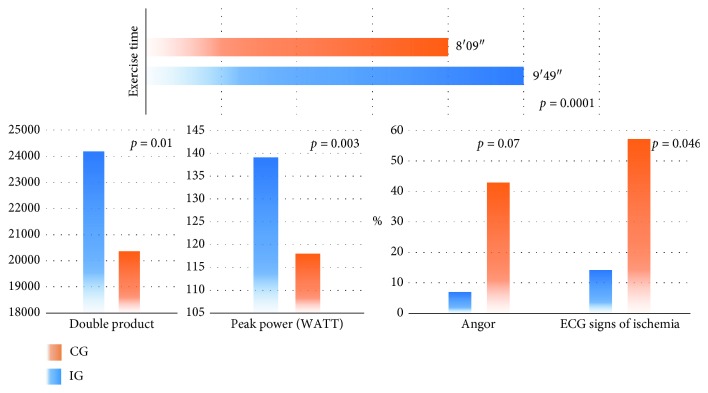
Stress test differences between control group (CG-orange) and ivabradine group (IG-blue) after 30 days of therapy.

Twenty-eight patients met inclusion criteria and were included in our study. After signing the informed consent, 14 patients were randomized in IG and other 14 patients in CG. The baseline clinical characteristics are shown in Table 1, and these are similar between two groups except for creatinine values, although these are in the normal range in all patients. The average age was 62.7 ± 8.8 years of patients assigned to IG and 61.8 ± 7.8 in CG, and the patients were mainly men (100% in IG and 86% in CG; *p*=0.48). The therapy at the randomization time was equally distributed in all patients (Table 2). As mentioned above, all patients underwent stress echocardiography at randomization time (T1), and the parameters, illustrated in Table 3, did not show any significant differences between two groups except for diastolic time at baseline but it was not confirmed at peak exercise (T1 baseline: IG 0.53 ± 0.03 msec vs CG 0.60 ± 0.09 msec *p*=0.01; peak: IG 0.74 ± 0.07 msec vs CG 0.67 ± 0.18 msec, *p*=0.2). The comparison about echocardiographic, electrocardiographic, and clinical parameters between two groups after 1 month is reported in Table 4. IG patients showed a significant reduction of heart rate at rest (IG from 71.1 ± 5.3 bpm to 63.6 ± 4.2 bpm *p*=0.0003) in contrast to CG (from 68.7 ± 8.1 bpm to 63.7 ± 10.5 bpm, *p*=0.17). HR at peak exercise was statistically higher in IG (HR max: IG 132.3 ± 13.2 bpm vs CG 120.4 ± 15 bpm, *p*=0.03) with a greater work load (IG 139.3 Watt vs CG 118.7 ± 19.6 Watt, *p*=0.003) and a significant longer exercise time (IG 9′49″ ± 48″ vs CG 8′09″ ± 59″; *p*=0.0001) (Figure 1). The metabolic equivalents expressed in METs were increased in IG (IG 10.4 ± 0.7 vs CG 9.3 ± 0.6, *p*=0.0001). Moreover, the use of ivabradine showed a better chronotropic reserve (IG 68.7 ± 14 vs CG 48.7 ± 13, *p*=0.0007). As expected, there were no differences in terms of systemic blood pressure whether at baseline or at peak exercise (baseline IG 122.8 ± 8 mmHg vs CG 120.7 ± 13 mmHg, *p*=0.6; peak IG 182.9 ± 8 mmHg vs CG 179.3 ± 20 mmHg, *p*=0.5). The incidence of angina, during the test, was lesser in IG than CG without statistical difference (7% vs 42.8%, *p*=0.07), but a lower rate of ST depression was observed with significant difference in IG (14.2% vs 57.1%, *p*=0.046). Rhythm alterations were not found. The myocardial O_2_ consumption expressed as DP and TP was better in IG (DP: IG 24194 ± 2697 vs CG 20358 ± 4671.8, *p*=0.01; TP : IG 17239 ± 4710 vs CG 12206 ± 4413, *p*=0.007) (Figure 1). The difference baseline/peak exercise of DP and TP was significantly greater in IG.

The echocardiographic parameters were collected in all patients, and no one was excluded for the thoracic impedance. After the exercise interruption, it was needed on average 2 minutes to record all data. The LVEF did not show a significant increase in both groups (IG 53.6 ± 2.2 vs CG 50.4 ± 6.1, *p*=0.07). The end diastolic diameter (EDD), before starting the exercise after 30 days of treatment, was lesser in IG (47.1 ± 2.3 vs 50.3 ± 4.0, *p*=0.01). At peak exercise, the ET corrected for HR was diminished in both groups, but there was a greater variation in IG than CG (0.76 ± 0.10 vs 0.58 ± 0.10, *p*=0.0001); on the contrary, the diastolic time (corrected for HR) was similar in both groups. The analysis of diastolic function after the test revealed an increase of E wave and A wave velocities, but the E/A ratio did not show a relevant difference. The max E′ wave velocity was greater in IG than in CG, and in the same group, the differences between baseline and peak exercise were greater (ΔE′ 3.14 ± 0.7 vs 2.4 ± 1.13, *p*=0.047). The E/E′ ratio was significant reduced in patients treated with ivabradine (IG 10.2 ± 2.0 vs CG 7.9 ± 1.6, *p*=0.002).

At 1 month follow-up, the incidence of angina episode was higher in CG, and we did not find rehospitalizations, cardiac death, myocardial infarction, and stroke.

## 4. Discussion

The key results of our study are as follows: the use of ivabradine in addition to standard therapy (1) improves the exercise tolerability, reducing the angina symptoms and the incidence of ST modifications, reaching a greater workload without sign or symptoms of ischemia, and (2) allows to perform a longer exercise with greater chronotropic reserve; (3) in IG there was an improvement in parameters related to the diastolic function during exercise; and (4) after one month of therapy, ivabradine seems to improve the ventricular remodeling due to ischemic disease.

Although IG showed a significant reduction of heart rate at rest, the HR was not significantly different than the CG after 30 days of therapy; therefore, the demonstrated clinical benefit did not seem to be related only to this parameter.

After therapy with ivabradine, we observed an increase of the ejection time (corrected for HR) and consequently the effective share of ejective systole with respect to the cardiac cycle. In several studies, ejection time was shown to be related to the left ventricular systolic function [12, 13].

The increase of the ejection time is accompanied by an increase of the triple product, which is closely related to the tension-time index, a measure of ventricular work and oxygen demand that is found by multiplying the average pressure in the ventricle during the period in which it ejects blood by the time it takes to do this [10].

Patients treated with ivabradine also showed better capacity to increase the heart rate with exercise or other metabolic demands (chronotropic reserve) even if they had a resting HR similar to the CG patients after 30 days of therapy.

The greater and longer stress tolerance in IG could be explained by the greater ability to increase HR and by the greater share of the cardiac cycle occupied by ejection time, as well as by an improvement of the diastolic function.

If in our sample the effects of ivabradine were not closely related to differences in resting HR, it must be considered that many pleiotropic effects of ivabradine are described by small studies in the literature [14, 15].

During ischaemia or heart failure, the normally low expression of hyperpolarization-activated cyclic nucleotide-gated channels (which carry the If current) outside the sinus node is increased [16].

It is useful to consider that beta blockers, unlike ivabradine, when properly titrated, reduce chronotropic reserve, inotropism, and lusitropism. In addition, especially when no beta 1-selective drugs are used, they expose alpha-adrenergic receptors during effort with a consequent increase in coronary resistance [17, 18].

In CONTROL-2 Study, conducted on patients with stable angina, the combination of therapy with ivabradine and *β*-blocker demonstrated good tolerability, safety, and more pronounced clinical improvement, compared to *β*-blocker up-titration after 16 weeks of follow-up [8].

Somehow, our data also support these results, but we must consider that the use of *β*-blockers, unlike ivabradine, has clearly demonstrated an improvement in long-term outcome in patients with ischemic heart disease. Therefore, a longer follow-up study would be useful to understand if the benefits of beta blockers are maintained even when these are not up-titrated in combination with ivabradine.

The echocardiographic data of diastolic function measured during exercise, before and after treatment, showed that ivabradine has an effect on protodiastolic relaxation (increasing of E′ velocity) and on the left ventricular diastolic filling pressure (significative reduction of E/E′ in IG). As suggested from our data, ivabradine had no effect on the diastolic times but seems to improve the quality of diastolic function. The small size of the sample did not allow us to use as a benchmark the classification of diastolic dysfunction in degrees, as described in the recommendations of the American Society of Echocardiography and the European Association of Cardiovascular Imaging 2016. However, our data show that some variables (which reflect filling pressures and left ventricular compliance) have an improving trend in the ivabradine group.

According to the results, we have to recognize how ivabradine was able to slow down the process of left ventricular remodeling in terms of LV diameters, compared to the control group. In previous studies, ivabradine has been shown to reduce oxidative stress in the myocardial wall and circulating angiotensin II levels with decreased plasma levels of IL-6, tumor necrosis factor-*α*, and norepinephrine [19–25]. Factors listed above are known as mediators able to influence cardiac structural remodeling and diastolic function.

## 5. Conclusions

In patients with residual myocardial ischemia after PCI, the addition of ivabradine to standard therapy improves ischemic threshold and reduces angina, with an increase of chronotropic reserve, double product, ejection time, and triple product, regardless HR. The addition of ivabradine resulted in a better diastolic function during exercise by stress-echocardiography evaluation and seems to prevent the ventricular remodeling.

## Figures and Tables

**Table 1:** Baseline clinical characteristics in ivabradine group (IG) and control group (CG).

	IG *N*=14	CG *N*=14	*p* value
Men (%)	100 (14)	86 (12)	0.48
Age (years)	62.7 ± 8.8	61.8 ± 7.8	0.76
Caucasian (%)	100 (14)	100 (14)	1
Previous myocardial infarction (%)	14 (2)	14 (2)	1
Previous angina (%)	14 (2)	43 (6)	0.20
Previous PCI (%)	14 (2)	14 (2)	1
Previous CABG (%)	0	0	—
Systemic hypertension (%)	43 (6)	71 (10)	0.25
Smoke (%)	71 (10)	57 (8)	0.69
Familial history of CAD (%)	100 (14)	86 (12)	0.48
Dyslipidemia (%)	57 (8)	43 (6)	0.70
Diabetes mellitus (%)	0	29 (4)	0.09
Peripheral artery disease (%)	0	0	—
Previous stroke (%)	0	0	—
Renal impairment (%)	0	0	—
Creatinine (mg/dl)	0.94 ± 0.15	0.85 ± 0.05	0.04^*∗*^
Previous bleeding (%)	0	0	
Multivessel coronary artery disease (%)	43 (6)	50 (7)	0.96

PCI = percutaneous coronary intervention; CABG = coronary artery bypass graft; CAD = coronary artery disease.

**Table 2:** Therapy of the ivabradine group (IG) and the control group (CG).

	IG *N*=14	CG *N*=14	*p* value
Aspirin (%)	86 (12)	86 (12)	1
Clopidogrel (%)	14 (2)	14 (2)	1
Prasugrel (%)	14 (2)	14 (2)	1
Ticagrelor (%)	71 (10)	71 (10)	1
Nitrates (%)	86 (12)	86 (12)	1
*β*-blockers (%)	100 (14)	100 (14)	1
Ca-antagonist (%)	0	14 (2)	0.48
ACE inhibitor (%)	71 (10)	86 (12)	0.64
Digoxin (%)	0	0	
ARBs (%)	29 (4)	14 (2)	0.64
Statins (%)	100 (14)	86(12)	0.48
Antiarrhythmic (%)	0	0	

ACE inhibitor = angiotensin-converting enzyme inhibitor; ARBs = angiotensin receptor blockers.

**Table 3:** Stress echocardiography at randomization time (T1).

	IG *n*=14 pts		CG *n*=14 pts			
	T1		T1		*p* value	
	Rest	Peak exercise	Rest	Peak exercise	Rest	Peak exercise
Mean heart rate (bpm)	71.1 ± 5.3	126.6 ± 11.1	68.7 ± 8.1	118.4 ± 17	0.36	0.14
Max HR (bpm)	75.4 ± 8.3		68.3 ± 12.2		0.08	
Chronotropic reserve (bpm)	53 ± 9		46 ± 14		0.12	
Exercise time	8′:15″±18.9″		7′:50″±47″		0.12	
METS	9.0 ± 0.3		8.6 ± 0.7		0.06	
Maximal test (%)	14%		14%		1	
DP	22255 ± 4504		19668 ± 4462		0.14	
ΔP	13.5 ± 3.5		11.11 ± 3.3		0.07	
Peak power (Watt)	121.4 ± 9.4		112.5 ± 13.7		0.06	
Symptoms (%)	57.1		42.8		0.70	
ECG alterations (%)	57.1		71		0.70	
Mean rest LVEF (%)	51.8 ± 2.9		51.7 ± 8.5		0.97	
LVEDD (mm)	48.1 ± 2.5	48.6 ± 2.1	49.9 ± 4.9	49.3 ± 4.2	0.98	0.97
LVEDV (ml)	133.6 ± 22.2	125.3 ± 19.2	126.9 ± 45.8	112.4 ± 39.5	0.62	0.28
LVESV (ml)	64.6 ± 12.1	57.8 ± 9.7	65.9 ± 32.4	54.7 ± 26.9	0.88	0.68
E wave (cm/sec)	63.3 ± 10.2	84.1 ± 6.6	70.4 ± 22.3	98.1 ± 34.6	0.28	0.14
A wave (cm/sec)	67.1 ± 15.4	83.6 ± 24.6	74.6 ± 14.0	95.6 ± 9.0	0.18	0.09
E/A	0.98 ± 0.23	1.13 ± 0.58	0.95 ± 0.3	1.01 ± 0.3	0.76	0.49
E′ wave (cm/sec)	8 ± 2.2	9.8 ± 1.55	7.0 ± 2.2	10.7 ± 3.5	0.24	0.38
E/E′	8.3 ± 1.91	8.7 ± 1.57	10.4 ± 3.4	9.7 ± 3.0	0.30	0.27
ET correct for HR (s)	0.47 ± 0.04	0.64 ± 0.14	0.45 ± 0.11	0.57 ± 0.11	0.52	0.15
DT correct for HR (s)	0.53 ± 0.03	0.74 ± 0.07	0.60 ± 0.09	0.67 ± 0.18	0.01^*∗*^	0.2
TP	14709 ± 5731		10916 ± 4451		0.06	

DP = double product; TP = triple product; LVEF = left ventricular ejection fraction; LVEDD = left ventricular end-diastolic diameter; ET = ejection time; DT = diastolic time; LVEDV = left ventricular end-diastolic volume; LVESV = left ventricular end-systolic volume.

**Table 4:** Stress echocardiography after 30 days of therapy (T2).

	IG *n*=14 pts		CG *n*=14 pts			
	T2		T2		*p* value	
	Rest	Peak exercise	Rest	Peak exercise	Rest	Peak exercise
Mean heart rate (bpm)	63.6 ± 4.2	132.3 ± 13.2	63.7 ± 10.5	120.4 ± 15	0.97	0.03
Max HR (bpm)	79.3 ± 7.1		71.1 ± 10.9		0.02^*∗*^	
Chronotropic reserve (bpm)	68.7 ± 14		0.0007^*∗*^		48.7 ± 14	
Systolic blood pressure (mmHg)	122.8 ± 8	182.9 ± 8	120.7 ± 13	179.3 ± 20	0.6	0, 5
Exercise time	9′:49″±48″		8′:09″±59″		0.0001^*∗*^	
METs	10.4 ± 0.7		9.3 ± 0.6		0.0001^*∗*^	
Maximal test (%)	30%		14%		0.06	
DP	24194 ± 2697		20358 ± 4671.8		0.01^*∗*^	
ΔDP	16.4 ± 2.8		12.6 ± 3.5		0.003^*∗*^	
Peak power (Watt)	139.3 ± 13.4		118.7 ± 19.6		0.003^*∗*^	
Symptoms (%)	7		42.8		0.07	
ECG alterations (%)	14.2		57.1		0.046^*∗*^	
Mean rest LVEF (%)	53.6 ± 2.2		50.4 ± 6.1		0.07	
LVEDD (mm)	47.1 ± 2.3	46 ± 2.5	50.3 ± 4.0	49.4 ± 3.6	0.01^*∗*^	0.07
LVEDV (ml)	129.7 ± 21.0	122.7 ± 18.5	128.4 ± 42.3	116 ± 34.9	0.15	0.53
LVESV (ml)	60.3 ± 10.2	55.9 ± 8.8	65.7 ± 30.3	57.6 ± 23.6	0.53	0.8
E wave (cm/sec)	65.0 ± 7.4	84 ± 7.9	70.9 ± 20.3	97.1 ± 29.6	0.31	0.12
A wave (cm/sec)	61.7 ± 14.2	80.4 ± 20.6	74.6 ± 11.9	91.1 ± 9.2	0.01^*∗*^	0.08
E/A	1.10 ± 0.3	1.14 ± 0.5	0.96 ± 0.3	1.06 ± 0.3	0.22	0.61
E′ wave (cm/sec)	7.4 ± 1.7	10.6 ± 1.9	7.1 ± 1.6	9.6 ± 2.4	0.6	0.2
E/E′	9.2 ± 2.7	7.9 ± 1.6	10.0 ± 2.4	10.2 ± 2.0	0.4	0.002^*∗*^
ΔE′	3.14 ± 0.7		2.4 ± 1.13		0.047^*∗*^	
ET corrected for HR (sec)	0.43 ± 0.04	0.76 ± 0.10	0.43 ± 0.90	0.58 ± 0.10	1	0.0001^*∗*^
DT corrected for HR (sec)	0.51 ± 0.05	0.78 ± 0.09	0.59 ± 0.09	0.76 ± 0.16	0.007^*∗*^	0.7
TP	17239 ± 4710		12206 ± 4413		0.007^*∗*^	
ΔTP	4785 ± 671		3359 ± 941		0.0001^*∗*^	

DP = double product; TP = triple product; LVEF = left ventricular ejection fraction; LVEDD = left ventricular end-diastolic diameter; ET = ejection time; DT = diastolic time.

## Data Availability

The raw data, descriptive statistics, and inferential statistics used to support the findings of this study are included within the article.

## Additional Points

The size of our sample limits the possibility of drawing certain conclusions. The duration of follow-up is too short to fully show the effects of the therapy, particularly with regard to cardiac remodeling and the improvement of left ventricular ejection fraction.
